# Metatranscriptome Analysis Deciphers Multifunctional Genes and Enzymes Linked With the Degradation of Aromatic Compounds and Pesticides in the Wheat Rhizosphere

**DOI:** 10.3389/fmicb.2018.01331

**Published:** 2018-07-03

**Authors:** Dhananjaya P. Singh, Ratna Prabha, Vijai K. Gupta, Mukesh K. Verma

**Affiliations:** ^1^ICAR-National Bureau of Agriculturally Important Microorganisms, Maunath Bhanjan, India; ^2^Department of Bio-Medical Engineering and Bio-Informatics, Chhattisgarh Swami Vivekanand Technical University, Bhilai, India; ^3^ERA Chair of Green Chemistry, Department of Chemistry and Biotechnology, School of Science, Tallinn University of Technology, Tallinn, Estonia

**Keywords:** metatranscriptome, xenobiotics, pesticides, polyaromatic hydrocarbons, polychlorinated biphenyls, petroleum hydrocarbons, microbial communities

## Abstract

Agricultural soils are becoming contaminated with synthetic chemicals like polyaromatic compounds, petroleum hydrocarbons, polychlorinated biphenyls (PCBs), phenols, herbicides, insecticides and fungicides due to excessive dependency of crop production systems on the chemical inputs. Microbial degradation of organic pollutants in the agricultural soils is a continuous process due to the metabolic multifunctionalities and enzymatic capabilities of the soil associated communities. The plant rhizosphere with its complex microbial inhabitants and their multiple functions, is amongst the most live and dynamic component of agricultural soils. We analyzed the metatranscriptome data of 20 wheat rhizosphere samples to decipher the taxonomic microbial communities and their multifunctionalities linked with the degradation of organic soil contaminants. The analysis revealed a total of 21 different metabolic pathways for the degradation of aromatic compounds and 06 for the xenobiotics degradation. Taxonomic annotation of wheat rhizosphere revealed bacteria, especially the Proteobacteria, actinobacteria, firmicutes, bacteroidetes, and cyanobacteria, which are shown to be linked with the degradation of aromatic compounds as the dominant communities. Abundance of the transcripts related to the degradation of aromatic amin compounds, carbazoles, benzoates, naphthalene, ketoadipate pathway, phenols, biphenyls and xenobiotics indicated abundant degradation capabilities in the soils. The results highlighted a potentially dominant role of crop rhizosphere associated microbial communities in the remediation of contaminant aromatic compounds.

## Introduction

Toxic organic compounds such as polychlorinated biphenyls (PCBs), chlorophenols, polycyclic aromatic hydrocarbons (PAHs), petroleum hydrocarbons, dyes and pesticides are becoming major contaminants of agricultural soils (Diez, 2010; Valentìn et al., 2013). Polyaromatic, chlorinated and nitrobenzene compounds, dioxins, PCBs and the pesticides represent principal xenobiotics (Sinha et al., 2009; Agrawal and Dixit, 2015) that cause biospheric pollution (Rabinovich et al., 2004). PAHs and PCBs are resistant organic contaminants against biodegradation by native microflora (Chikere, 2013; Bisht et al., 2015). Crop roots and the rhizosphere are directly under the influence of hardly biodegradable organic pollutants and contaminants, especially the PAHs and pesticides under any contaminated soil environment (Chen et al., 2013; Lawal, 2017). This may create serious and threatening concerns for the healthy soil and crop environment and therefore, the biodegradation and/or rhizoremediation of the chemical wastes and organic pollutants are now attracting central focus worldwide (Chikere, 2013; Lu et al., 2014; Kronenberg et al., 2017).

The chemicals when used continuously, increasingly and excessively in the cropping system become contaminants raising the problems of soil and rhizosphere pollution (Mwangi et al., 2010; Chauhan and Singh, 2015). Abundance of organic contaminants and pesticides in the food chain and water is reported to cause carcinogenesis, neurotoxicity and reproduction and cell development disorders (Burrows et al., 2002; Damalas and Eleftherohorinos, 2011; Prüss-Ustün et al., 2011; Myers et al., 2016). Therefore, the process of biodegradation or biologically catalyzed transformation of organic contaminants is of prime importance (Karigar and Rao, 2011; Joutey et al., 2013; Kehinde and Isaac, 2016). Although abiotic pathways are known for the breakdown of organic pollutants and pesticides in the soils and water, microbe-mediated degradation is the primary mechanism of contaminant remediation and detoxification (Singh and Walker, 2006; Akbar and Sultan, 2016; Javaid et al., 2016). The role of microbial communities is of immense importance due to their multifunctional degradation capabilities (Das and Chandran, 2011; Dechesne et al., 2014). Microbial (bacterial and fungal) degradation of pesticide, xenobiotic compounds and organic chemicals has been widely reported (McGuinness and Dowling, 2009; Porto et al., 2011) since long, although the direct, indirect or intermediary linking of the metabolic pathways connected with the removal of contaminants is less understood (Ladino-Orjuela et al., 2016).

Rhizosphere represents the plant-root interface which is populated by distinctive microbial communities having a functional influence on the plants (McNear, 2013). In the rhizosphere, potential communities accelerate biodegradation processes and help plants enhance co-metabolism to degrade organic contaminants by (i) facilitating selective enrichment of biodegrader microorganisms for degradation of xenobiotics in the root free soils (Nichols et al., 1997) (ii) enhancing metabolism of microbial growth by secreting natural substrate depending on the quantity of xenobiotics (Haby and Crowley, 1996; Alexander, 1999) and (iii) enriching natural compounds that can provoke co-metabolism of xenobiotics in specific microbes exhibiting genes or plasmids with degradation functions (Gupta et al., 2016). In any way, crop rhizosphere resists or tolerates to the level of organic contaminants, xenobiotics and pesticides, majorly with the help of microbial functions linked with the metabolic degradation pathways that partially or totally detoxify or minimize quality and quantity of contaminants in the soils. Various studies mentioned microbial biodegradation of different PCBs and xenobiotics through plant roots (Pilon-Smits, 2005; Balcom et al., 2016) along with genes involved in degradation (Wittich, 1998; Arai et al., 2000; Duffner et al., 2000; Esteve-Núñez et al., 2001; Furukawa et al., 2004; Galvão et al., 2005) and transcriptional regulators responsible for degradation of aromatic compounds (Uchiyama and Miyazaki, 2013).

Metagenomics and metatranscriptomics approaches that allowed exploration of taxonomic communities and help linking of associated functions without cultivation of microorganisms (Schloss and Handelsman, 2003) specifically facilitated overall examination of microbial community-linked metabolic pathways related to specific metabolic functions. The catabolic genes related to the degradation of xenobiotics can be characteristically annotated and linked to the identified taxonomic group/genus of the microbial communities (Chikere, 2013; Sharma and Sharma, 2018). We have annotated and deciphered twenty wheat rhizosphere metatranscriptomes available from EBI Metagenomics database for digging out transcripts/genes related to direct degradation or intermediary degradation functions of aromatic compounds and xenobiotics and reported prospective rhizoremediation capacity linked with the rhizobiome of the crop plants.

## Materials and Methods

### Metatranscriptome Datasets

Twenty wheat rhizosphere metatranscriptome samples (ERS025421, ERS238568, ERS238569, ERS238570, ERS238571, ERS238572, ERS238573, ERS238574, ERS238575, ERS238576, ERS238577, ERS238578, ERS238579, ERS238580, ERS238581, ERS238582, ERS238583, ERS238584, ERS238585, and ERS238586) were downloaded from the EBI Metagenomics database^1^. Raw sequence reads were taken for each dataset and processed further for the annotation and analysis.

### Quality Check and Metatranscriptome Sample Processing

Raw sequence reads of each sample were processed separately and were uploaded to the MG-RAST (Metagenome Rapid Annotation using Subsystem Technology) server^2^ (Meyer et al., 2008). Further processing was carried out through the standard pipeline keeping default parameters. For identification and annotation of proteins and other sequences, sequence similarity searches were done against different databases associated with MG-RAST pipeline. Genbank (Benson et al., 2013, 2015), protein databases M5NR (Wilke et al., 2012), SEED (Chen et al., 2013), and Kyoto Encyclopedia of Genes and Genomes (KEGG) (Kanehisa et al., 2008) were used for annotations at various levels. The data analysis was performed using High Performance Supercomputing Facility of ICAR-National Bureau of Agriculturally Important Microorganisms, India.

### Taxonomic and Functional Annotation

For identification of taxonomic abundance, all the datasets were individually aligned against M5NR database (Wilke et al., 2012) and Genbank (Benson et al., 2013, 2015) through the MG-RAST pipeline (Meyer et al., 2008). Parameters taken include maximum *E*-value: 1 × 10^−5^, minimum percentage identity: 60%, and minimum alignment length: 15. For the purpose of functional annotation, datasets were aligned against SEED subsystems taking into account similar parameters (Overbeek et al., 2014; Imchen et al., 2017).

### Metabolic Pathway Analysis

For identification and functional characterization of diverse metabolic pathways related to the metabolism of aromatic compounds and xenobiotics biodegradation and metabolism, SEED Subsystems was applied from the MG-RAST pipeline (parameters were minimum alignment length 15 and *E*-value cutoff 1e-5). For obtaining entire metabolic pathways information, global gene expression was annotated and further visualized with KEGGmapper (an in-built tool on the basis of the KEGG pathway mapping system). Level 2 and Level 3 SEED subsystem of MG-RAST was applied for annotation. Level 3 of SEED subsystem corresponds to KEGG pathway^3^. Furthermore, the Enzyme Commission (EC) number was identified for the sequences of corresponding enzymes. Pathways, transcripts and enzymes for the biological processes were identified in each dataset and corresponding abundance of transcripts/enzymes was calculated.

### Availability of Data and Associated Information

The metatranscriptome dataset are publically available from the EBI Metagenomics database^4^.

## Results and Discussion

Organic chemical contaminants, toxic wastes and synthetic pesticides are reported to damage the quality of agricultural soils and disturb the intrinsic balance between biological and ecological functional services (Valentìn et al., 2013). Microbial communities with their efficient metabolic pathways and enzymatic capabilities help breakdown of contaminating organic chemicals and pesticides through biotransformation and biodegradation (Parte et al., 2017). The functions of microorganisms pertaining to biotransformation and biodegradation help improve soil quality under the conditions of xenobiotic contamination (Ortiz-Hernández et al., 2013). The metatranscriptome analysis reports such widely abundant microbial communities, which are known for their role of degradation and remediation of organic pollutants and prominently abundant transcripts, which were directly related to the enzymes that have potential to degrade various organic chemicals in 20 wheat rhizosphere metatranscriptomes.

### Assessment of Taxonomic Abundance

**FIGURE 1:**
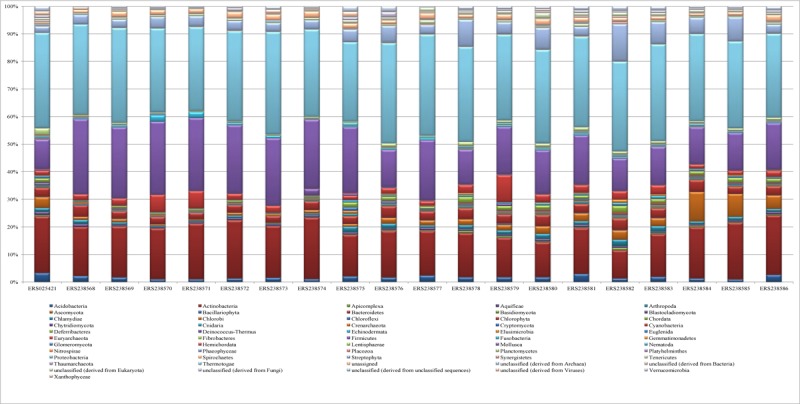
The abundance of different phyla in the 20 rhizosphere metatranscriptome samples.

A total of 688.8 Mb cDNA datasets varying in size from 2.8 to 102.3 Mb and reads from 6,832 to 249,64720 of 20 wheat rhizosphere metatranscriptomes were analyzed. Bacterial communities dominated taxonomic profiling in all the samples followed by eukaryota. Within the identified bacterial phylum, Proteobacteria and Actinobacteria remained the most dominant phyla. Firmicutes, Bacteroidetes, Streptophyta, Cyanobacteria, Acidobacteria, Verrucomicrobia, Planctomycetes, Ascomycota and unclassified microbes represented major phyla in wheat rhizosphere (**Figure 1**). Microbial degradation is reported to be the major process by which aromatic compounds are biodegraded/biotransformed into less toxic, persistent and complex metabolites (Haritash and Kaushik, 2009). The abundance of Proteobacteria, Actinobacteria, Acidobacteria, Bacteroidetes, Planctomycetes, and Firmicutes have earlier been reported from the soils (Janssen, 2006; Shade et al., 2012) and rhizosphere of many plants like *Arabidopsis* spp. (Lundberg et al., 2012; Bulgarelli et al., 2013; Schlaeppi et al., 2014), oak (Uroz et al., 2010), potato (Inceoglu et al., 2011), cucumber (Tian and Gao, 2014), soybean (Mendes et al., 2014), rice (Edwards et al., 2015), wheat (Ai et al., 2015; Donn et al., 2015), maize (Garcia-Salamanca et al., 2013; Peiffer et al., 2013) and others (Pascual et al., 2016).

Proteobacteria, being extremely diverse phylum comprising chemolithoautotrophs, heterotrophs and mixotrophs (Chao, 1984; Bent and Forney, 2008) have been extensively reported from different ecological zones in which they manage and regulate biogeochemical cycles (Ivarsoon and Holm, 2008). These communities are common inhabitant of soils to help functions for carbon, nitrogen and sulfur cycling (Spain et al., 2009; Castañeda and Barbosa, 2017; Yang et al., 2017). Their dominant abundance of the Proteobacteria as the largest phylum in the rhizosphere may possibly be attributed to their rapid growth rate (Johnston-Monje et al., 2016) and may have functional significance for the wheat in maintaining nutrient needs of the crop. The phyla Proteobacteria remained dominated by alpha-, beta- and gamma-proteobacterial communities that occupy diazotrophic status and possess symbiotic ability, root association and endophytic colonization with the plants (Pisa et al., 2011). Alpha-proteobacteria including the genera Sphingomonas and Azospirillum and gamma-proteobacteria, Xanthomonas remained dominant communities for the degradation of PAHs (Vinas et al., 2005; Simarro et al., 2013; Bisht et al., 2015). The study thus, indicated the dominance of diverse microbial communities, which were known for improved rhizoremediation against chemical pollutants in the soils contaminated with PAH, and other compounds (Kuiper et al., 2004).

The other dominant community of the wheat rhizosphere was the Actinobacteria, which are involved in the carbon cycle and secondary metabolite production (Jenkins et al., 2010; Castañeda and Barbosa, 2017). The phyla include different species with significant ecological importance and roles in decomposition, humus formation, nitrogen fixation and antibiotic production (Bond et al., 2004; Barka et al., 2016; Kielak et al., 2016). These communities are known biodegraders of PAHs (Leticia, 2006; Ghosal et al., 2016), pesticides (De Schrijver and De Mot, 1999; Fuentes et al., 2010), heavy metals (Alvarez et al., 2017), and xenobiotics (Polti et al., 2014). Firmicutes that also represented dominant community in the wheat rhizosphere inhabited varied range of environment as anaerobes (e.g., Clostridia) and aerobes (e.g., *Bacillus*). The phylum Bacteroidetes present in the wheat rhizosphere comprised of aerobes, anaerobes and facultative anaerobes (Janssen, 2006) that hold multifunctional potential in the soils (Wang et al., 2016). We also identified sequences linked with the cyanobacteria, the photosynthetic autotrophs commonly inhabiting agricultural soils (Oliveira et al., 2017). Cyanobacteria have also been predominantly implicated in the biodegradation of pesticides (Subramanian et al., 1994; Ibrahim et al., 2014), textile dyes (Dellamatrice et al., 2017) and aromatic compounds (Semple et al., 1999).

### Aromatic Metabolism Pathway

**FIGURE 2:**
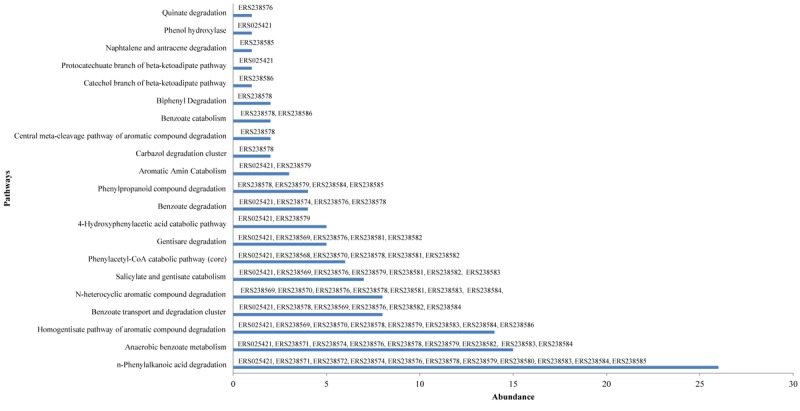
Abundance of pathways related to the metabolism of aromatic compounds in wheat metatranscriptomes. Name of the samples in which genes/enzymes were identified are written over the abundance of each pathway.

Analysis of wheat rhizosphere metatranscriptomes for the pathways and transcripts related to the metabolism and degradation of aromatic compounds led to the identification of multiple enzymes associated with 21 different pathways (**Table 1**). Pathways identified for the metabolism of aromatic compounds were aromatic amin catabolism, Benzoate transport and degradation cluster, Gentisare degradation, Phenylacetyl-CoA catabolic pathway (core), Carbazol degradation cluster, Anaerobic benzoate metabolism, 4-Hydroxyphenylacetic acid catabolic pathway, Catechol branch of beta-ketoadipate pathway, Central meta-cleavage pathway of aromatic compound degradation, Homogentisate pathway of aromatic compound degradation, N-heterocyclic aromatic compound degradation, Protocatechuate branch of beta-ketoadipate pathway, Salicylate and gentisate catabolism, Benzoate catabolism, Benzoate degradation, Biphenyl Degradation, Naphtalene and anthracene degradation, Phenol hydroxylase and Phenylpropanoid compounds, Quinate and *n*-Phenylalkanoic acid degradation (**Figure 2**). Overall, 118 transcripts of 47 different enzymes linked with 21 pathways of aromatic compound metabolism were identified. The enzyme 2-hydroxy-6-oxo-6-phenylhexa-2,4-dienoate hydrolase is commonly present in different pathways of aromatic metabolism namely carbazole degradation cluster, central meta-cleavage pathway of aromatic compound and biphenyl degradation. Members of this enzyme family unusually catalyze hydrolysis of C-C bonding in the biphenyl degradation pathway (Horsman et al., 2006). There were abundant (8) transcripts linked with the enzyme isoquinoline 1-oxidoreductase alpha subunit (EC 1.3.7.16) in the dataset for *N*-heterocyclic aromatic compound degradation pathway. The enzyme is specific for the degradation of N-heterocyclic compounds like isoquinoline and quinazoline and was reported from alphaproteobacteria *Brevundimonas diminuta*.^5^

**Table 1:** Identified aromatic degradation pathways and genes/enzymes involved in the metabolism of aromatic compounds in wheat rhizosphere metatranscriptome samples.

Pathway	Genes/Enzymes
Aromatic amin catabolism	4-hydroxyphenylacetate 3-monooxygenase (EC 1.14.13.3),
	Monoamine oxidase (1.4.3.4)
Benzoate transport and degradation cluster	4-oxalocrotonate decarboxylase (EC 4.1.1.77)
	Benzoate transport, inner-membrane translocator
	Benzoate transport, inner-membrane translocator precursor
	Benzoate-CoA ligase (EC 6.2.1.25)
	Benzoyl-CoA oxygenase component B
	Predicted 2-keto-4-pentenoate hydratase/2-oxohepta-3-ene-1,7-dioic acid hydratase
	Shikimate kinase I (EC 2.7.1.71)
Benzoate catabolism	Benzoate 1,2-dioxygenase beta subunit (EC 1.14.12.10)
	Muconolactone isomerase (EC 5.3.3.4)
Benzoate degradation	Benzoate 1,2-dioxygenase alpha subunit (EC 1.14.12.10)
	Benzoate 1,2-dioxygenase beta subunit (EC 1.14.12.10)
	Ortho-halobenzoate 1,2-dioxygenase alpha-ISP protein OhbB
	benzoate dioxygenase, ferredoxin reductase component
Anaerobic benzoate metabolism	2-hydroxycyclohexanecarboxyl-CoA dehydrogenase (EC 1.1.1.-)
	3-hydroxybutyryl-CoA dehydrogenase (EC 1.1.1.157)
	Acetyl-CoA acetyltransferase (EC 2.3.1.9)
	Benzoate-CoA ligase (EC 6.2.1.25)
	Glutaryl-CoA dehydrogenase (EC 1.3.99.7)
Carbazol degradation cluster	2-hydroxy-6-oxo-6-phenylhexa-2,4-dienoate hydrolase (EC 3.7.1.-)
Homogentisate pathway of aromatic compound degradation	4-hydroxyphenylpyruvate dioxygenase (EC 1.13.11.27)
	Fumarylacetoacetase (EC 3.7.1.2)
	Homogentisate 1,2-dioxygenase (EC 1.13.11.5)
	Maleylacetoacetate isomerase (EC 5.2.1.2)
	Transcriptional regulator, IclR family
Gentisare degradation	Fumarylacetoacetate hydrolase family protein
	Maleylacetoacetate isomerase (EC 5.2.1.2)
Salicylate and gentisate catabolism	Fumarylacetoacetase (EC 3.7.1.2)
	Fumarylacetoacetate hydrolase family protein
	Maleylacetoacetate isomerase (EC 5.2.1.2)
*n*-Phenylalkanoic acid degradation	3-hydroxyacyl-CoA dehydrogenase (EC 1.1.1.35)
	3-ketoacyl-CoA thiolase (EC 2.3.1.16)
	Enoyl-CoA hydratase (EC 4.2.1.17)
	Long-chain-fatty-acid–CoA ligase (EC 6.2.1.3)
Phenylacetyl-CoA catabolic pathway (core)	Phenylacetate-CoA oxygenase, PaaG subunit
	Phenylacetate-CoA oxygenase, PaaH subunit
	Phenylacetate-CoA oxygenase/reductase, PaaK subunit
	Phenylacetic acid degradation protein PaaD, thioesterase
	Phenylacetic acid degradation protein PaaE, ketothiolase
	Phenylacetic acid degradation protein PaaN, ring-opening
	aldehyde dehydrogenase (EC 1.2.1.3)
4-Hydroxyphenylacetic acid catabolic pathway	2-hydroxyhepta-2,4-diene-1,7-dioate isomerase (EC 5.3.3.-)
	4-hydroxyphenylacetate 3-monooxygenase (EC 1.14.13.3)
	5-carboxymethyl-2-oxo-hex-3- ene-1,7-dioate decarboxylase (EC 4.1.1.68)
Protocatechuate branch of the beta-ketoadipate pathway	3-carboxy-*cis,cis*-muconate cycloisomerase (EC 5.5.1.2)
Catechol branch of the beta-ketoadipate pathway	Muconolactone isomerase (EC 5.3.3.4)
Central meta-cleavage pathway of aromatic compound degradation	2-hydroxy-6-oxo-6-phenylhexa-2,4-dienoate hydrolase (EC 3.7.1.-)
Phenylpropanoid compound degradation	Chlorogenate esterase
	Phenylpropionate dioxygenase and related ring-hydroxylating
	dioxygenases, large terminal subunit
	Transcriptional regulator for ferulate or vanillate catabolism
	Vanillate *O*-demethylase oxygenase subunit (EC 1.14.13.82)
Phenol hydroxylase	Phenol hydroxylase, P3 oxygenase component DmpN (EC 1.14.13.7)
Biphenyl degradation	2-hydroxy-6-oxo-6-phenylhexa-2,4-dienoate hydrolase (EC 3.7.1.-)
*N*-heterocyclic aromatic compound degradation	Isoquinoline 1-oxidoreductase alpha subunit (EC 1.3.99.16)
Biphenyl Degradation	2-hydroxy-6-oxo-6-phenylhexa-2,4-dienoate hydrolase (EC 3.7.1.-)
Naphtalene and anthracene degradation	2-hydroxychromene-2-carboxylate isomerase
Quinate degradation	3-dehydroquinate dehydratase I (EC 4.2.1.10)

Various other pathways for aromatic compound degradation were also identified in the dataset. We identified transcripts for central meta-cleavage pathway, using which many organisms degrade high quantity of phenol (Mahiudddin et al., 2012). Besides, transcripts linked with homogentisate pathway and *N*-heterocyclic aromatic compound degradation was also identified. 2-hydroxy-6-oxo-6-phenylhexa-2,4-dienoate hydrolase was identified for the central meta-cleavage pathway of aromatic compound degradation. Similarly, transcript for the isoquinoline 1-oxidoreductase alpha subunit (EC 1.3.99.16) was detected in the dataset for *N*-heterocyclic aromatic compound degradation (**Table 1**). For naphtalene and anthracene degradation, the transcripts related to the enzyme 2-hydroxychromene-2-carboxylate isomerase was also detected. The enzyme 2-hydroxychromene-2-carboxylate isomerase is known to degrade naphthalane sulfonates and anthracene and has been reported from bacterial species *Pseudomonas testosteroni* A3 and *Sphingomonas yanoikuyae* B1 (Kuhm et al., 1993; Kim et al., 2006).

#### Aromatic Amin Catabolism

The transcripts related to two prominent enzymes namely 4-hydroxyphenylacetate 3-monooxygenase (EC 1.14.13.3) and monoamine oxidase (1.4.3.4) were detected in wheat rhizosphere metatranscriptomes (**Table 1**). The enzyme 4-hydroxyphenylacetate 3-monooxygenase is involved in the breakdown of the aromatic ester 4-hydroxyphenylacetate, a ubiquitous compound that acts as the intermediate of aromatic amino acids, lignin and styrene (Mohamed et al., 2002). Members of this enzyme were reported from *Escherichia coli* (Xun and Sandvik, 2000). Monoamine oxidase family of flavoproteins catalyzes oxidative degradation of primary and secondary amines including polyamines, amino acids and tyramine (Díaz et al., 2001; Gaweska and Fitzpatrick, 2011). The cursing ecological role of polyclic and heterocyclic aromatic amines can be understood from the fact that this class of chemicals are carcinogens and environmental toxicants (Turesky and Le Marchand, 2011). Aromatic amines are also the component of polymers, dyes and other industrial products. Pesticide-derived aromatic amines commonly enter into the soil ecosystem following chemical control of insects and weeds in corn, soybean and other crops (Koutros et al., 2009; Luciana et al., 2015). Therefore, the presence of transcripts linked with the enzymes for the catabolism of aromatic amines signifies their role in the probable rhizoremediation of wheat rhizosphere.

#### Benzoate Degradation and Catabolism

Benzoate is a model compound to study anaerobic catabolism of aromatic compounds (Barragán et al., 2012). Benzoate is converted to benzoyl-Co-A following aerobic and anaerobic degradation pathways (Shinoda et al., 2004). Bacterial degradation of benzoate in the environment reflects biological strategy of microbial communities to increase cell fitness against these contaminants (Valderrama et al., 2012). Amongst the aromatic compounds and xenobiotic degradation linked transcripts found in this analysis, those related to the enzymes for degradation of benzoate were the most abundant in the wheat rhizosphere metatranscriptomes (**Figure 2**). Different enzyme families like decarboxylase, ligase, oxygenase, hydratase, kinase, isomerase, reductase, inner membrane translocater and alpha and beta subunits of dioxygenase were identified for the benzoate metabolism pathways including benzoate transport, degradation cluster and catabolism (**Figure 2** and **Table 1**). Linked to the anaerobic benzoate degradation, we identified 15 sequences related to different enzymes including Acetyl-CoA acetyltransferase (EC 2.3.1.9). The enzyme was abundantly detected in 9 rhizosphere samples along with Benzoate-CoA ligase (EC 6.2.1.25) and Glutaryl-CoA dehydrogenase (EC 1.3.99.7), both of which were traced in two samples (**Figure 2**). Acetyl-CoA acetyltransferase enzyme participates in the benzoate degradation via CoA ligation and is also involved in the metabolic pathways including those of aromatic amino acid metabolism^6^. Aerobic degradation of benzoate is also reported in different microbial communities including gram-negative proteobacterium *Azoarcus evansii* and gram-positive *Bacillus stearothermophilus* (Zaar et al., 2001).

**FIGURE 3:**
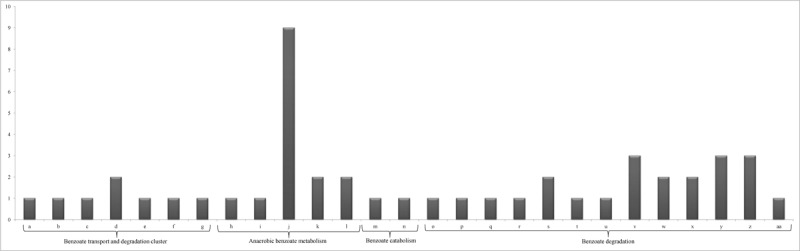
Abundance of transcripts related to benzoate degradation identified through KEGG analysis of the wheat rhizosphere metatranscriptome datasets. [a: 4-oxalocrotonate decarboxylase (EC 4.1.1.77); b: Benzoate transport, inner-membrane translocator; c: Benzoate transport, inner-membrane translocator precursor; d: Benzoate-CoA ligase (EC 6.2.1.25); e: Benzoyl-CoA oxygenase component B; f: Predicted 2-keto-4-pentenoate hydratase/2-oxohepta-3-ene-1,7-dioic acid hydratase; g: Shikimate kinase I (EC 2.7.1.71); h: 2-hydroxycyclohexanecarboxyl-CoA dehydrogenase (EC 1.1.1.-); i: 3-hydroxybutyryl-CoA dehydrogenase (EC 1.1.1.157); j: Acetyl-CoA acetyltransferase (EC 2.3.1.9); k: Benzoate-CoA ligase (EC 6.2.1.25); l: Glutaryl-CoA dehydrogenase (EC 1.3.99.7); m: Benzoate 1,2-dioxygenase beta subunit (EC 1.14.12.10); n: Muconolactone isomerase (EC 5.3.3.4); o: Benzoate 1,2-dioxygenase alpha subunit (EC 1.14.12.10); p: Benzoate 1,2-dioxygenase beta subunit (EC 1.14.12.10); q: benzoate dioxygenase, ferredoxin reductase component; r: Ortho-halobenzoate 1,2-dioxygenase alpha-ISP protein OhbB; s: 2,3-dihydroxybenzoate decarboxylase (EC:4.1.1.46); t: benzoate-CoA ligase (EC:6.2.1.25); u: benzoate/toluate 1,2-dioxygenase subunit beta (EC:1.14.12.10 1.14.12.-); v: benzoate/toluate 1,2-dioxygenase electron transfer component; w: muconolactone D-isomerase (EC:5.3.3.4); x: 2-pyrone-4,6-dicarboxylate lactonase (EC:3.1.1.57); y: 4-oxalmesaconate hydratase (EC:4.2.1.83); z: 3-carboxy-*cis,cis*-muconate cycloisomerase (EC:5.5.1.2); aa: 4-carboxymuconolactone decarboxylase (EC:4.1.1.44)].

Seven different enzymes for benzoate transport and degradation cluster were detected in the rhizosphere samples including benzoate inner membrane translocator and its precursor (**Figure 3**). Benzoate-CoA ligase, which was identified in two samples (ERS025421 and ERS238578) is the initial enzyme of benzoate metabolism in anaerobic organism *Geobacter metallireducens* (Wischgoll et al., 2005). Furthermore, the enzymes/transcripts for benzoate 1,2-dioxygenase beta subunit (EC 1.14.12.10) and muconolactone isomerase (EC 5.3.3.4) were also detected in the samples ERS238578 and ERS238586, respectively (**Figure 3**). Benzoate 1,2-dioxygenase catalyzes aromatic ring hydroxylation (Yamaguchi and Fujisawa, 1980) while the enzyme muconolactone isomerase involves in the catabolism of catechol to succinate (Katti et al., 1989). The alpha and beta subunits of benzoate 1,2-dioxygenase (EC 1.14.12.10 and EC 1.14.12.10), benzoate dioxygenase, ferredoxin reductase component and ortho-halobenzoate 1,2-dioxygenase alpha-ISP protein OhbB were also traced in the metatranscriptome samples. All these enzymes contribute to the degradation of benzoates in the environment significantly and therefore, their abundance in the wheat rhizosphere indicates their rhizoremedial significance.

#### Carbazole Degradation

Two transcripts for the enzyme 2-hydroxy-6-oxo-6-phenylhexa-2,4-dienoate hydrolase (HOPD hydrolase) related to the carbazole degradation were identified in the wheat metatranscriptome (**Figure 2**). Degradation of carbazole, the *N*-heterocyclic aromatic compound, members of which occupy components of petroleum products is reported by dioxygenation, a process majorly implicated by Proteobacterial and Actinobacterial phyla (Salam et al., 2017). Many bacterial species possessing these genes degrade PCBs (Masai et al., 1997). Owing to these reasons, carbazole degradation is considered as the remediation strategies for rhizosphere soils using bacterial species (Yang et al., 2009).

#### Salicylate, Gentisare and Quinate Pathway

Salicylate and gentisate are recognized as the intermediates in the naphthalene metabolism (Balachandran et al., 2012). We report high abundance of the transcripts (05) linked with the gentisare degradation, followed by those of salicylate and gentisate catabolism (07) and quinate degradation (01). We have also detected high abundance of transcripts (14) linked with the enzymes of homogentisate pathway, a central catabolic pathway for the degradation of phenylalanine, tyrosine and hydroxyphenylacetic acid in microorganisms including *Pseudomonas putida* (Arias-Barrau et al., 2004). The transcripts related to the enzymes fumarylacetoacetase, fumarylacetoacetate hydrolase family protein, maleylacetoacetate isomerase and 3-dehydroquinate dehydratase were detected in multiple metatranscriptomes for salicylate, gentisare and quinate metabolism. Apart from these, the enzymes homogentisate 1,2-dioxygenase and 4-hydroxyphenylpyruvate dioxygenase linked with the homogentisate pathway of aromatic compound degradation were also detected (**Table 1**). The enzymes fumarylacetoacetase hydrolase and maleylacetoacetate isomerase catalyze hydrolytic breakdown of fumarylacetoacetate and metabolic degradation of phenylalanine and tyrosine (Polekhina et al., 2001). Homogentisate, after its formation from phenylalanine and tyrosine through hydroxylation is further catabolized to fumerate and acetoacetate via three enzymes homogentisate dioxygenase (HmgA), fumarylacetoacetate hydrolase (HmgB) and maleylacetoacetate isomerase (HmgC). Interestingly, the transcripts related to all these three enzymes were detected in the dataset, reflecting an evidence of utilization of homogentisate compounds by the native wheat rhizosphere microbial communities. In the soils, salicylate, quinate and gentisare are supposed to be derived from the organic matter residues of plant sources (Livermore et al., 2014). In the terrestrial habitat where the plant leaf litter and other decaying organic debris are consistently available as carbon source, the abundance of such transcripts linked with the potential enzymes having capability to degrade phenylalanine and tyrosine probably indicates more conserved carbon pathways of the native microbial communities.

#### *n*-Phenylalkanoic Acid Degradation

The esters of poly-phenylalkanoates (PHAs) are novel microbial biodegradable plastics usually formed from 3-hydroxy-*n*-phenylalkanoic acids (Garcia et al., 1999). Under nutrient limiting conditions but with the availability of excess carbon sources, PHAs are accumulated in the microorganisms either in the culture conditions or in the natural habitats like soil and water. The transcripts related to the enzymes 3-hydroxyacyl-CoA dehydrogenase (EC 1.1.1.35), 3-ketoacyl-CoA thiolase (EC 2.3.1.16), Enoyl-CoA hydratase (EC 4.2.1.17) and Long-chain-fatty-acid–CoA ligase (EC 6.2.1.3) that degrade PHAs are most abundantly (26) present in the wheat rhizopshere metatranscriptome samples (**Table 1**). PHAs are known to be produced intracellular by bacteria and archea as storage carbon and energy source (Koller et al., 2011). It can be inferred that after the decay of the bacterial cells with PHAs as storage compounds, living microbial communities may find access of these compounds under unfavorable conditions of nutrient limitations. The dominance of the transcripts related to PHA degrading enzymes in the wheat rhizosphere metatranscriptomes is supposed to enable associated microbial communities maintain the rhizospheric ecosystem through carbon recycling (Ray and Kalia, 2017).

#### Phenylacetyl-CoA Catabolic Pathway

Phenylacetyl-CoA, a component of various substrates like phenylalanine, lignin-related phenylpropane and phenylalkanoic acid is amongst the major environmental contaminants (Teufel et al., 2010) and intermediate product of microbial degradation (Díaz et al., 2001). This is also an effector molecule of the TetR family regulator isolated from the extreme thermophilic bacterium *Thermus thermophilus* HB8 and is supposed to be involved in phenylacetic acid degradation (Sakamoto et al., 2011). Various genes/enzymes such as Phenylacetate-CoA oxygenase (PaaG subunit), Phenylacetate-CoA oxygenase (PaaH subunit), Phenylacetate-CoA oxygenase/reductase (PaaK subunit), Phenylacetic acid degradation protein PaaD (thioesterase), Trancripts related to phenylacetic acid degradation protein PaaE, PaaN, ketothiolase and ring-opening aldehyde dehydrogenase (EC 1.2.1.3) were identified for phenylacetyl-CoA catabolic pathway in the metatranscriptome samples (**Table 1**). In the dataset, transcripts linked with the enzymes for 4-hydroxyphenylacetic acid catabolic pathway (2-hydroxyhepta-2,4-diene-1,7-dioate isomerase, 4-hydroxyphenylacetate 3-monooxygenase and 5-carboxymethyl-2-oxo-hex-3-ene-1,7-dioate decarboxylase were also detected (**Table 1**). Since phenylacetic acid is among the common intermediate in the pathway of structurally similar aromatic compounds, the presence of the transcripts linked with its degradation reflects the remediation capability of wheat rhizosphere microbial communities. Abundance of these transcripts is indication of microbial community-linked functions involved in phenylacetyl-CoA metabolism.

#### Beta-Ketoadipate Pathway

The beta-ketoadipate pathway is a chromosomally encoded convergent pathway involved in degradation of aromatic compounds and is in general present in soil bacteria and fungi (Harwood and Parales, 1996). Transcripts for two different branches of this pathway, i.e., catechol branch of the beta-ketoadipate pathway (muconolactone isomerase, EC 5.3.3.4) and protocatechuate branch of the beta-ketoadipate pathway (3-carboxy-*cis,cis*-muconate cycloisomerase, EC 5.5.1.2) were identified in the rhizosphere metatranscriptome dataset (**Table 1**). The β-ketoadipate pathway (β-KAP) is involved in the conversion of hazardous aromatic pollutants into simple metabolites like tricarboxylic acid. Studies have also shown that this pathway for conversion of aromatic lignocellulosic waste into bio-oils can be used for producing biodiesel (Wells and Ragauskas, 2012).

#### Phenylpropanoid and Biphenyl Pathway

Phenols and related phenolic compounds are major organic pollutants causing severe damage to the environment (Diez, 2010). Phenols also adversely affect microbial taxa richness, which can influence the degradation of other pollutants (Barrios-Martinez et al., 2006; Cordova-Rosa et al., 2009). Phenol hydroxylase is reported from many bacterial species to catalyze the catabolism of phenol (Silva et al., 2013). A transcript related to the enzyme phenol hydroxylase, P3 oxygenase component DmpN was identified in the metatranscriptome reflecting that wheat rhizosphere possesses microbial communities for rhizoremedial significance (Nordlund et al., 1993; Kirchner et al., 2003). Also transcripts for the enzymes linked with the phenylpropanoid compound degradation were also identified. Chlorogenate esterase, henylpropionate dioxygenase and related ring-hydroxylating dioxygenases, large terminal subunit, transcriptional regulator for ferulate or vanillate catabolism and vanillate *O*-demethylase oxygenase subunit were traced in the metatranscriptome. Apart from this, the transcript for related to the enzyme 2-hydroxy-6-oxo-6-phenylhexa-2,4-dienoate hydrolase responsible for biphenyl degradation was also identified in the wheat rhizosphere. Besides industrial source of phenolic compounds, a major source of phenolics in the agricultural soils are the plants and native organisms. Presence of transcripts for phenolics degrading enzymes suggests that the microbial communities in the wheat rhizosphere may potentially degrade these compounds as their source of carbon and energy. PCBs are persistent pollutants and their aerobic degradation by soil microorganisms involves biphenyl and benzoate pathways (Leewis et al., 2016).

### Pathways for Xenobiotics Degradation

Apart from the identification of transcripts/enzymes in the pathways involved in the metabolism of aromatic compounds in wheat rhizosphere metatranscriptomes, we have also explored the transcripts and pathways linked with the xenobiotic degradation and metabolism. Overall, 37 transcripts of 16 different enzymes were identified from 6 pathways linked with the degradation of xenobiotic compounds (**Table 2**). Connected with the degradation of xenobiotics, six pathways and their associated enzymes were identified in 9 samples (ERR263035, ERR031114, ERR263026, ERR263028, ERR263029, ERR263033, ERR263031, ERR263036, and ERR263034) with varied levels of abundance (**Table 2**). These pathways were linked with the degradation of chlorocyclohexane, chlorobenzene, benzoate, aminobenzoate, nitrotoluene, styrene and caprolactam. Among both of these metabolic processes, i.e., metabolism of aromatic compounds and xenobiotic degradation, benzoate degradation pathways was commonly identified.

**Table 2:** Identified active pathways, genes/enzymes and their abundance in xenobiotics degradation in wheat rhizosphere metatranscriptome samples.

Pathway	Genes/Enzymes	Abundance	Sample metatranscriptome samples
Chlorocyclohexane and chlorobenzene degradation	E1.14.13.7; phenol 2-monooxygenase [EC:1.14.13.7]	9	ERR263035,
			ERR031114,
	E3.1.1.45; carboxymethylenebutenolidase [EC:3.1.1.45]		ERR263026,
			ERR263028,
			ERR263029,
			ERR263033
Benzoate degradation	DHBD; 2,3-dihydroxybenzoate decarboxylase [EC:4.1.1.46]	18	ERR031114,
			ERR263028,
	badA; benzoate-CoA ligase [EC:6.2.1.25]		ERR263029,
			ERR263031,
			ERR263033,
	benB-xylY; benzoate/toluate 1,2-dioxygenase subunit beta [EC:1.14.12.10 1.14.12.-]		ERR263036
	benC-xylZ; benzoate/toluate 1,2-dioxygenase electron transfer component		
	catC; muconolactone D-isomerase [EC:5.3.3.4]		
	ligI; 2-pyrone-4,6-dicarboxylate lactonase [EC:3.1.1.57]		
	ligJ; 4-oxalmesaconate hydratase [EC:4.2.1.83]		
	pcaB; 3-carboxy-*cis,cis*-muconate cycloisomerase [EC:5.5.1.2]		
	pcaC; 4-carboxymuconolactone decarboxylase [EC:4.1.1.44]		
Aminobenzoate degradation	mdlC; benzoylformate decarboxylase [EC:4.1.1.7]	5	ERR263035,
			ERR031114
	vanA; vanillate monooxygenase [EC:1.14.13.82]		
Nitrotoluene degradation	E1.12.99.6L; hydrogenase large subunit [EC:1.12.99.6]	2	ERR263026,
			ERR263036
Styrene degradation	E3.5.5.7; aliphatic nitrilase [EC:3.5.5.7]	2	ERR263033
Caprolactam degradation	E1.14.13.22; cyclohexanone monooxygenase [EC:1.14.13.22]	1	ERR263034

Analysis of the datasets further revealed that transcripts linked with the benzoate degradation genes/enzymes were high in abundance (**Table 2**). Enzymes like 2,3 dihydrobenzoate decarboxylate (DHDB) that reversibly catalyzes decarboxylation of 2,3-dihydroxybenzoate into catechol in *Clostridium hydroxybenzoicum* (He and Wiegel, 1996), benzoate-CoA ligase (badA) from *Magnetospirillum* sp. strain TS-6 that initially catalyzes benzoate degradation (Kawaguchi et al., 2006) and benB-xylZ-beta subunit of benzoate/toluene 1,2-dioxygenase that catalyze 1,2-dihydroxylation of different benzoate variants (Haddad et al., 2001) were detected (**Figure 3**).

We have also detected electron transfer gene component of the enzyme benzoate 1,2-dioxygenase encoded by benC as has been reported by Neidle et al. (1991). Similarly, catC; muconolactone D-isomerase which was traced in the metatranscriptome dataset was also identified from *P. putida*.^7^ Interestingly, the dataset showed transcript matching with the enzymes like ligI; 2-pyrone-4,6-dicarboxylate lactonase (**Figure 3**) that catalyzes the degradation of protocatechuates, methylgallate and syringate in *Pseudomonas* species^8^, ligJ; 4-oxalmesaconate hydratase from *Sphingomonas* sp. SKY6^9^ and pcaB-3-carboxy-*cis,cis*-muconate cycloisomerase and pcaC-4-carboxymuconolactone decarboxylase, muconate lactonizing enzymes in microorganisms responsible in dehalogenation of muconate derivatives of haloaromatic xenobiotic compounds (Kajander et al., 2002) (**Table 2**). These results indicated the presence of diverse microbial communities and functions linked with the degradation capabilities of aromatic compounds and xenobiotics, which may otherwise have negative impact on the crop plants. Our analysis further supplements earlier observations (Kuiper et al., 2004; Thomas and Cébron, 2016) that suggested the role of rhizosphere microbial inhabitants in rhizoremediation for plant and soil benefits. Rhizosphere of crops being highly rich in diversity of microbial communities are supposed to be extremely metabolically active and multifunctional (Li et al., 2014). This study shows rich diversity of microbial phyla at both the taxonomic and functional level. The significance of the abundance of functional genes/transcripts in the wheat metatranscriptome samples may possibly be marked as an adaptation strategy of soil microbial communities in the rhizosphere in the presence of toxic aromatic compounds including pesticides (Xu et al., 2013).

While analyzing the datasets for identification of pathways related to xenobiotics degradation and metabolism, transcripts for the enzymes involved in the degradation of chlorocyclohexane, chlorobenzene, nitrotoluene, styrene and caprolactam were also identified (**Table 2**). Chlorobenzene causes narcosis, tremors, restlessness, and muscle spasms in animals after inhalation while in humans, its chronic or long term exposure influences the central nervous system (CNS)^10^. Styrene, on exposure, causes eye irritation, gastrointestinal effects, headache, fatigue, weakness, and depression etc. in humans, depending on the duration of exposure^11^. Similarly, caprolactam also influences the human health in a negative way on exposure^12^. Looking into the harmful ecological perspectives of the xenobiotics, this study has practical significance as it identifies diversely abundant taxonomic phyla in the wheat rhizosphere and relates identified transcripts to the enzymes linked with the metabolic pathways known for the organic contaminant metabolism or degradation in the soils.

Overall, the dominance of function-linked microbial communities in the rhizosphere of wheat further signifies their role in rhizoremediation of organic pollutants around the plant roots, an area which needs high attention under the conditions of increasing anthropogenic interactions belowground. The study also indicates that rhizosphere microbial communities possess capabilities to utilize the materials like PHAs produced and accumulated by microorganisms themselves under nutrient limiting conditions and deprived state of carbon and energy. The communities also degrade diverse phenylpropanoids introduced to the soils as the debris of leaf litter from plants for the source of carbon. Thus, the metatranscriptome analysis reflected both the pollutant remediation role for the microbial communities associated with such functions as well as the ecological role of maintaining the carbon cycle within the rhizosphere.

## Conclusion

Identification of genes and enzymes involved in the metabolism and degradation of organic compounds and xenobiotics can serve as an efficient strategy for bioremediation approaches. Taxonomic exploration of the metatranscriptomes of wheat rhizosphere led to the identification of diverse taxonomic phyla having proven functional role in pollutant degradation. Genes, enzymes and pathways involved in degradation of different organic pollutants like aromatic amines, benzoates, carbazoles, styrene, phenols and biphenols were identified. Detection of particular genes and enzymes involved in pesticide and xenobiotics degradation and their further utilization for bioremediation of the environmental niches, particularly those contaminated with aromatic hydrocarbons is an important aspect. For the biological degradation of aromatic compounds, xenobiotics and pesticides, it is critical to understand the molecular mechanisms engaged in enzymatic catalysis, which will make feasible designing of novel substitutes along with proficient tools for the treatment of pesticide residues and bioremediation of contaminated locations. The study reflected the dominance of taxonomic phyla abundance of transcripts and enzymes linked with the ecological functions of degradation and metabolism. It further reflects that because of the dominance of potential microbial functionalities that link with the metabolic degradation processes for detoxifying or minimizing the impacts of the contaminants in the soils, crop rhizosphere becomes able to tolerate and/or resist harmful organic chemicals. The information on presence of the dominant microbial communities in the rhizosphere obtained through non-cultivable approach may open avenues for the exhaustive isolation and characterization of potential species with the soil bioremediation functions.

## Author Contributions

RP and DS designed the study, performed the analysis and wrote the manuscript. VG and MV edited the manuscript and made modifications.

## Conflict of Interest Statement

The authors declare that the research was conducted in the absence of any commercial or financial relationships that could be construed as a potential conflict of interest.

## Footnotes

1 https://www.ebi.ac.uk/metagenomics/

2 http://metagenomics.anl.gov/

3 ftp://ftp.metagenomics.anl.gov/data/manual/mg-rast-tech-report-v3_r4.pdf

4 https://www.ebi.ac.uk/metagenomics/

5 http://www.uniprot.org/uniprot/Q51697

6 https://biocyc.org/META/NEW-IMAGE?type=PATHWAY&object=PWY-283

7 https://www.wikigenes.org/e/gene/e/1045925.html

8 https://biocyc.org/META/NEW-IMAGE?type=REACTION&object=RXN-2462

9 https://biocyc.org/gene?orgid=META&id=MONOMER-16015

10 https://www.epa.gov/sites/production/files/2016-09/documents/chlorobenzene.pdf

11 https://www.epa.gov/sites/production/files/2016-09/documents/styrene.pdf

12 https://www.epa.gov/sites/production/files/2016-09/documents/caprolactam.pdf
